# Eradicating dominant ideologies in higher education: the responsibility of campus leadership in developing a culturally-congruent education experience

**DOI:** 10.1080/1360080x.2024.2333594

**Published:** 2024-03-27

**Authors:** Jose H. Vargas, José M. Paez, Yolanda Vasquez-Salgado, Will Garrow, Carrie L. Saetermoe

**Affiliations:** a California State University, Northridge, CA, USA; b Health Equity Research and Education Center, Northridge, CA, USA

**Keywords:** Antiracism, cultural mismatch theory, dominant ideologies, educational leadership, intersectionally-marginalised students of colour, race/racism

## Abstract

Educational leadership serves a pivotal function in establishing the tenor of campus cultures. Executive decisions shape educational policy and practice in ways that either hinder or advance marginalised students’ academic success. Leaders are in powerful positions to modify unjust academic ecosystems and to de-ideologise the white-centric dominant ideologies that lead to student pushout. Leadership actions tend to justify the status quo rather than to deconstruct campus culture and reconstruct antiracist options. Inaction from leadership has resulted in cultural discontinuities that lead some students to self-deidentify from academia. This article expands upon *cultural mismatch theory* to describe how race dysconsciousness, academic capitalism and rugged individualism operate to buttress ecosystemic conditions that create racially-antagonistic campus cultures. We propose strategies that leaders can implement to eradicate dominant ideologies at their home institutions and within educator-student relationships and individual mindsets. We also discuss important considerations and corresponding actions for creating culturally-congruent equity-focused educational spaces.

[T]here arises a need to increase critical consciousness through a process of de-ideologization[.] What this involves is introducing into the ambience of the collective consciousness elements and schemata that can help dismantle the dominant ideological discourse and set in motion the dynamics of a process of de-alienation. — Ignacio Martín-Baró, 1994, p. 189

Educational racism structures all of higher education. Global movements against critical race theory and antiracist educational initiatives (see Kingkade et al., 2021; Miller et al., 2023), as well as momentous legal actions implicating higher education (e.g., the US Supreme Court overturning affirmative action, SFFA v. Harvard College, 2023), have worsened pre-existing academic inequities. As the US and other countries undergo demographic shifts that forecast national-level rises in ethnic/racial diversity (see Lomax et al., 2020; Vespa et al., 2020), institutional responsiveness to similar college-level trends lags behind. Educational spaces are complex ecosystems shaped by the divergent interests of individuals with differential access to power (see Bronfenbrenner, 1979; Martín-Baró, 1994). As such, individual and structural aspects of social life are fused (see Lerner, 2015). Lack of institutional preparation for impending postsecondary population shifts is traceable to enduring dominant ideologies and system-justifying discourses (see Vargas & Saetermoe, 2023). Predominantly-white institutions and majority-white academic pools are common ([NCES] National Center for Education Statistics, 2018; Hrabowski, 2012) and lead to hierarchical campus cultures that *pushout* students of colour (see Vargas et al., 2021), gender minorities (see Herrmann et al., 2016), working-class students (see Stephens, Fryberg, et al., 2012) and students who are intersectionally subordinated. Administrators, managers and senior staff shape educational policy and practice. Via their executive decisions, leaders are positioned to modify unjust academic cultures by combating white-centric dominant ideologies and advancing critical strategies that uplift the community cultural wealth of marginalised students (see Yosso, 2005).

We contend that intersectionally-marginalised students of colour in the US are pushed out of higher education via ecosystemic racism (see Vargas & Saetermoe, 2023) and institutionalised academic cultures that are in discord with marginalised communities’ values. The ecosystems of education perpetuate processes of *ideologisation*, whereby academics receive (and endorse) system-sustaining messages that are constructed and continually reinforced by a majoritarian and racist social order (see Martín-Baró, 1994). Educational leaders in the US tend to justify the status quo rather than to deconstruct campus culture and reimagine antiracist alternatives (see also Smith et al., 2021). System-justification is driven by mainstream culture’s norms and values (Vargas et al., 2021). The *pushout problem*, or the cultural processes within campuses that lead students to self-deidentify from academia, can be reframed as a corollary of three historically-unchallenged and stable dominant ideologies: (a) race dysconsciousness, (b) academic capitalism and (c) rugged individualism. Dominant ideologies sustain cultural discontinuities between white-centric standards and the customs/values of intersectionally-marginalised students (see also Vargas & Kemmelmeier, 2013). In line with Martín-Baró’s claim about challenging dominant ideologies, US educational leaders must play an active role in *de-ideologisation*, which refers to acts that help leaders, academics and students deconstruct the racist foundations of educational spaces and, in turn, co-reconstruct antiracist and liberatory environments. Although dominant ideologies differ by geographic region, nation and type of institution (e.g., public vs. private), a review of these nuances goes beyond the scope of this article. Still, international readers may find parallels to their own contexts. Thus, the aim of this article is three-fold. First, within a US context and in reference to higher education institutions in general, we examine the aforesaid dominant ideologies and outline linkages to ecosystemic racism. This analysis is couched within the cultural mismatch paradigm (Stephens, Fryberg, et al., 2012). Second, using vignettes and the extant research, we offer strategies leaders can employ to shift dominant ideologies and promote critical campus values. These ideological shifts address cultural mismatch and attenuate student pushout. Last, we discuss important considerations and corresponding actions for creating culturally-congruent equity-focused educational spaces.

## Dominant ideologies and cultural mismatch

Campus leadership can respond to dominant ideologies that maintain educational inequity through the institutionalisation of antiracist and culturally-congruent interventions. However, the comprehensive knowledge needed to eradicate these ideologies is rarely held by most educators (Vargas & Saetermoe, 2023). The ecosystemic quality and function of educational racism are hidden phenomena in need of exposure (see Vargas et al., 2021). Educational racism does not occur in a vacuum. Bronfenbrenner’s (1979) theory of ecological systems explains how human development is predicated on interconnected individual-, micro-, meso-, and macro-level events and social processes. The acquisition and internalisation of a person’s belief structure depend on interactions between families, peers, social institutions and culture (see Lerner, 2015). Similarly, Martín-Baró’s (1994) liberation psychology stresses co-creative processes between psyche and society. From this perspective, structural oppression and self-conceptualisation co-construct each other and, in turn, reproduce the social conditions that dehumanise, harm and alienate both the oppressed and the oppressor. To break oppression cycles, the social-political structures that keep dominant/subordinate persons in ideologised states must be unveiled, critiqued and transformed. In higher education, de-ideologisation begins with an awareness of the ecosystems wherein racialised ideologies and racial power operate.

### Ecosystemic racism and ideologies of racial power

**Figure 1.**
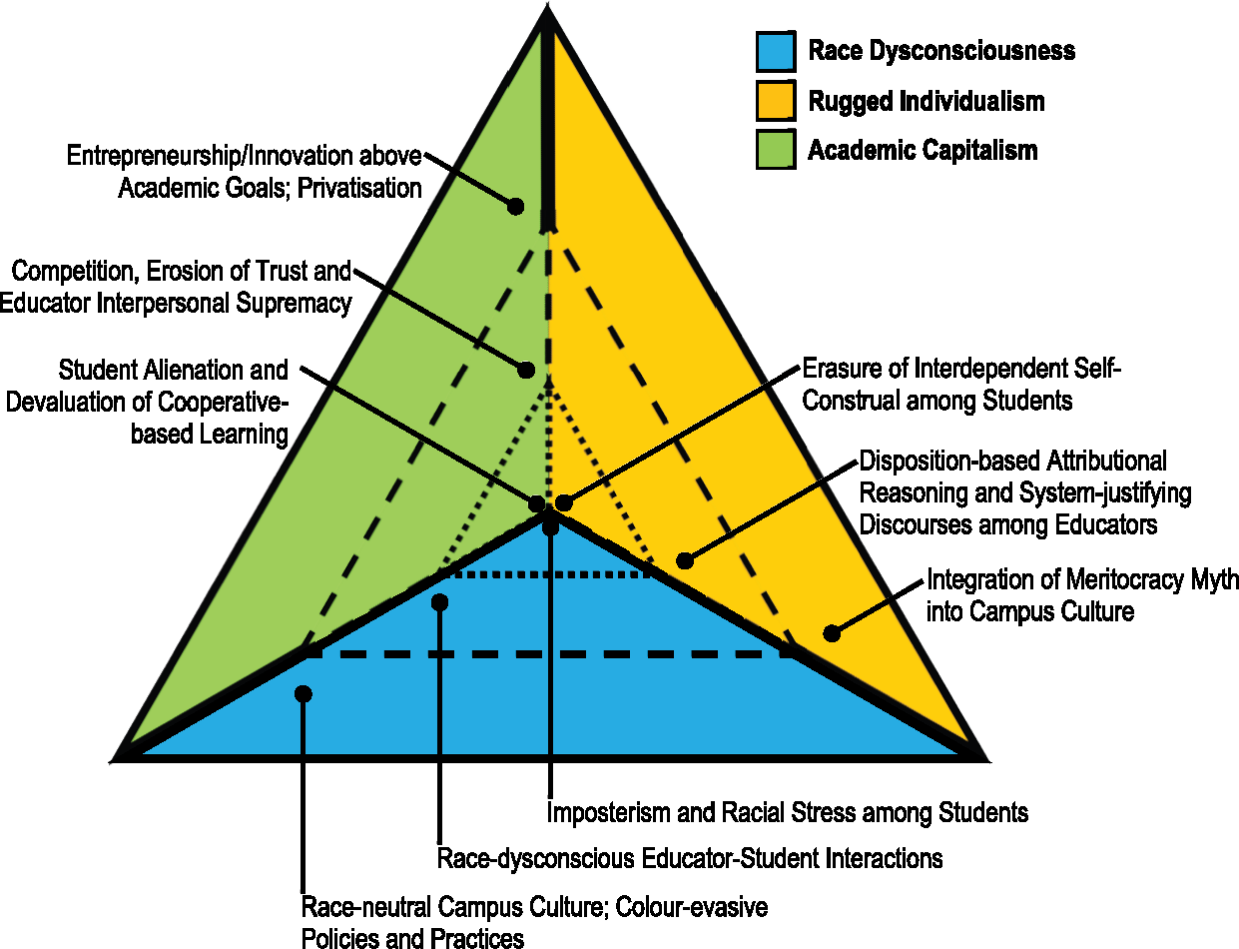
The three ecosystems of a hierarchical higher education institution. Inner Dotted Triangle: Psychological Level. Middle Dashed Triangle: Interpersonal Level. Outer Solid Triangle: Institutional Level.

Racism is a phenomenon that operates at the levels of individual actions (see Gaertner & Dovidio, 1986), entire social systems (see Bonilla-Silva, 1997, 2022a, 2022b) and throughout the bidirectional processes that fuse individual-systemic relations (see also Bronfenbrenner, 1979; Lerner, 2015; Martín-Baró, 1994; Vargas et al., 2021). Most educators are unaware of the individual-systemic relations that lead to injustice. In the journal *Educational Psychologist*, Vargas and Saetermoe (2023) coined the term *ecosystemic racism* to describe the ‘multilevel and recursive human events that implicate phenomenology, interpersonal transactions, local institutions, social-political structures, and intergenerational processes in the reproduction of race-based power hierarchies’ (p. 4). Contexts implicating race yield institutional, interpersonal and psychological effects in virtually all educational spaces. Dominant ideologies maintain a hierarchical social order by exploiting each of these levels (Figure 1). This manifests in the ways that higher education infuses mainstream societal values and false histories, in how educators pushout intersectionally-marginalised students of colour, and in the student experiences that cultivate self-doubt and imposterism. Next, we describe race dysconsciousness, academic capitalism and rugged individualism to show how these three dominant ideologies bolster educational racism at the institutional, interpersonal and intrapsychic levels.

### Race dysconsciousness

Ecosystemic racism mutates across time to adapt to and persist across the vicissitudes of social life (Vargas et al., 2021). Overt expressions of legal and extralegal racism – once key to the inception and continuance of settler-colonial nations – have fallen out of favour in previous decades (see Bonilla-Silva, 1997). Racism has become covert and integral to the formation of race-neutral and colour-evasive cultures (see Bonilla-Silva, 2022a). The decision in SFFA v. Harvard College (2023) illustrates the extent to which societies naively (or shrewdly) perceive value in race-neutrality and colour evasion despite their well-documented adverse consequences (see Bonilla-Silva, 2022b). A result of race-neutrality is the institutionalisation of colour-evasive policies and practices. Race-dysconscious institutions curb belongingness by sending messages to intersectionally-marginalised students of colour that their cultural backgrounds are not to be valued (see Saetermoe et al., 2017). Ecosystemic and liberation perspectives suggest that race-dysconscious institutions reinforce the oppressive social conditions that alienate students and enable their pushout from higher education (see Bronfenbrenner, 1979; Martín-Baró, 1994). Institutions dysconscious of ecosystemic racism adversely influence the interpersonal dynamics between educators and students. King (1991) advanced the idea of *dysconscious racism* to describe a mindset that uncritically accepts racist social orders. Race-dysconscious interpersonal transactions limit intersectionally-marginalised students of colour’s prospects when educators and leaders engage in avoidance, benevolent prejudice, denial discourses, microaggressions, system-justification and white saviorism (Vargas et al., 2021). Race-dysconscious institutions are ill-equipped to label and redress the social behaviours that uphold educational inequities (Vargas & Saetermoe, 2023). The psychological effects of race-dysconscious cultures for students are numerous and may include imposterism, racial stress and pushout (Vargas et al., 2021).

### Academic capitalism

In cultures like the US, educational systems are nested within the broader socioeconomic system of capitalism (see also Bronfenbrenner, 1979; Martín-Baró, 1994; Vargas et al., 2021). An effect of late-stage capitalism has been the increasing permeability between higher education and the economic market (Hanley, 2005). *Academic capitalism* refers to market and market-like actions undertaken in higher education to secure external funding sources and extract monetary value from the educational, research and service functions of institutions (Slaughter & Leslie, 2001). Under this ideology, campuses compete for prestige and limited resources while leaning on a market ethos of privatisation and individualism (Johnson & Taylor, 2019). As institutional leaders embrace academic capitalism, their efforts to promote innovation and entrepreneurship often threaten the academic aims of higher education (Croucher & Lacy, 2022). Extended control by leaders over academic production has also yielded negative consequences for academics and students (see McClure, 2016). Campus academics must compete for declining intramural monies and infrastructure. This constrains cooperation and how academics interact with marginalised students, resulting in forms of interpersonal supremacy that normalise uncritical pedagogies that obstruct trust (see also Vargas et al., 2021). Students are treated as cheap/free labour or as empty receptacles to be filled with impersonal knowledge. In exchange for campus rankings, reputation and secure revenue streams, academic capitalism creates alienating conditions that devalue cooperative-based education and push out intersectionally-marginalised students of colour.

### Rugged individualism

The US and similar Western nations are highly individualist (see Bellah et al., 1986; Martín-Baró, 1994). Hofstede (1980) described *individualism* as a culture-level orientation that prioritises the autonomy and self-interests of individuals. Individualism is also a personality-level phenomenon (see Singelis, 1994). The ‘rugged’ form of individualism emerged from the unique conditions associated with capitalism, Protestantism and frontier-style colonialism. This philosophy yielded mythical ideas about self-reliance, personal responsibility and individual achievement (see Bazzi et al., 2020). Likewise, cross-cultural social psychologists use the term ‘vertical individualism’ to describe an autonomous self that is defined by the personal successes obtained via competition (Vargas & Kemmelmeier, 2013). Leaders who draw from rugged or vertical individualism fuel dehumanising ‘sink-or-swim’ meritocracies. Further, interactions with intersectionally-marginalised students of colour may falter when educators lean on disposition-based attributions that individualise inequity and blame students for their own pushout (Vargas et al., 2021). Cultural discontinuities between independent and interdependent values work against nontraditional students (e.g., first-generation or working-class; see Stephens, Fryberg, et al., 2012), many of whom are students of colour. Erasure of interdependent selves – via campus cultures set by educational leadership – is academically detrimental.

### Cultural mismatch theory and the racist function of dominant ideologies

*Cultural mismatch theory* provides information about how universities might align their missions and actions with the values of diverse students (see Stephens, Fryberg, et al., 2012; Vasquez-Salgado et al., 2021). The theory posits that higher education institutions operate under white, middle-class and individualist norms. These norms are in misalignment with the collectivistic values of most first-generation and low-income students. Culturally-congruent interventions eliminate educational disparities (Stephens, Townsend, et al., 2012). Cultural mismatch theory illuminates the critical role institutions can play in addressing longstanding academic disparities. There is a need for institutions to modify their practices so that they align with the cultural norms of intersectionally-marginalised students of colour.

**Figure 2.**
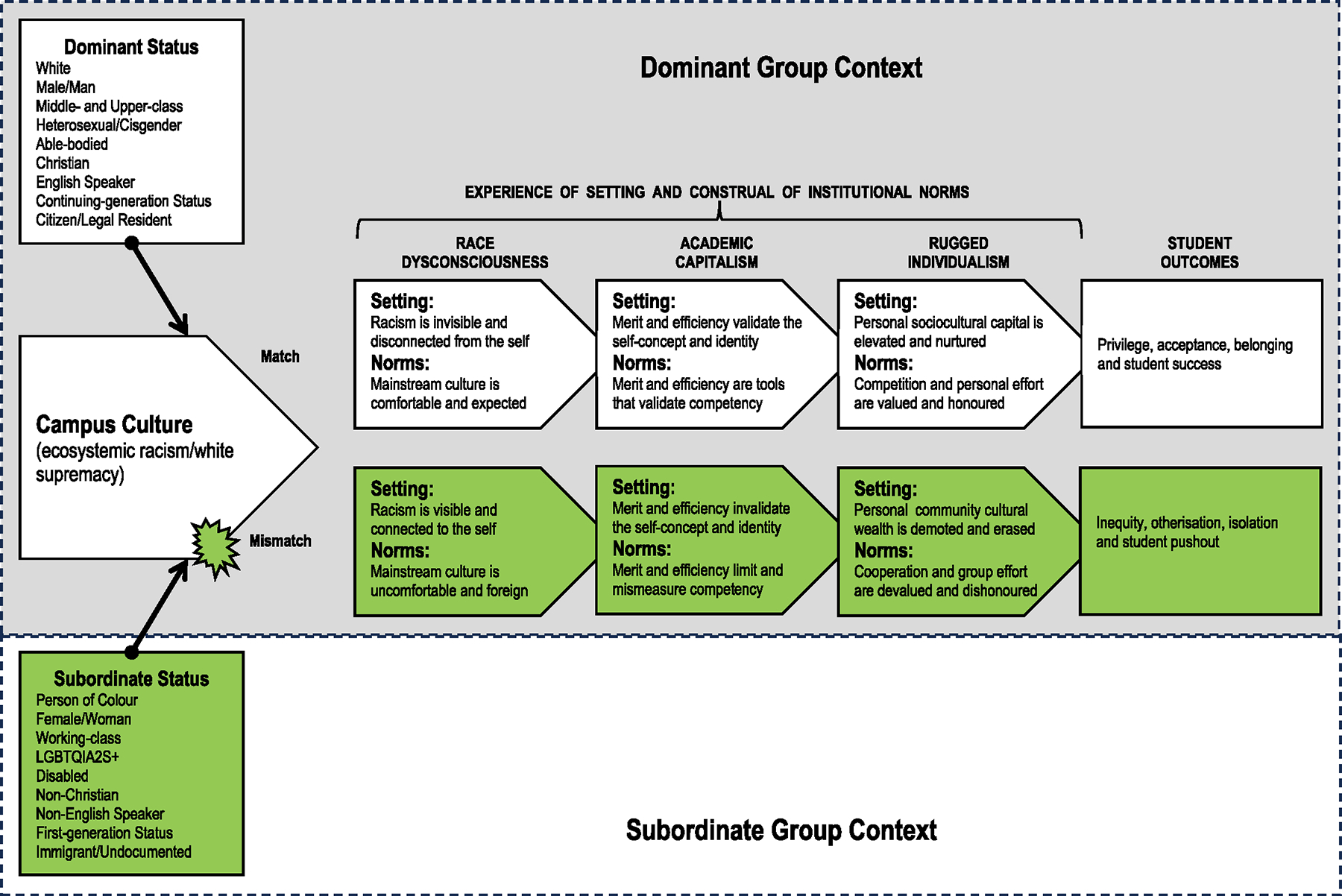
Modification of the cultural mismatch theory.

The application of cultural mismatch theory to the analysis of dominant ideologies may seem unorthodox given the theory’s focus on first- and continuing-generation student statuses (see Stephens, Fryberg, et al., 2012). We argue that cultural mismatch theory highlights a special case of more generalised forms of domination/subordination (Figure 2). Ideologies of oppression impact the ecosystems of social life (see also Bronfenbrenner, 1979; Martín-Baró, 1994). Ecosystemic racism and white supremacy intersect with other ecosystems of subordination (e.g., classism, sexism) to conserve social arrangements that favour dominant group members (see also Vargas & Saetermoe, 2023). Race dysconsciousness, academic capitalism and rugged individualism shape how educators and students experience their academic settings and how institutional norms are construed. For dominant group members, racism is invisible, merit-based education validates the self-concept and white-centric cultural capital is cherished. Meritocracy, colour evasion and competition are expected and honoured. Conversely, for subordinate group members, racism is visibly self-relevant, meritocracy threatens the self-concept and community cultural wealth is demoted or erased. Academia’s norms are uncomfortable, foreign, mismeasure competency and devalue cooperation. Thus, students whose cultural orientations align with the dominant group context experience privilege, acceptance, belonging and student success; for intersectionally-marginalised students of colour, cultural discontinuities lead to inequity, otherisation, isolation and pushout. Accordingly, leadership must take an active role in shifting dominant ideologies that prop up racist power in academia.

## Shifting dominant ideologies: from uncritical to critical leadership

**Figure 3.**
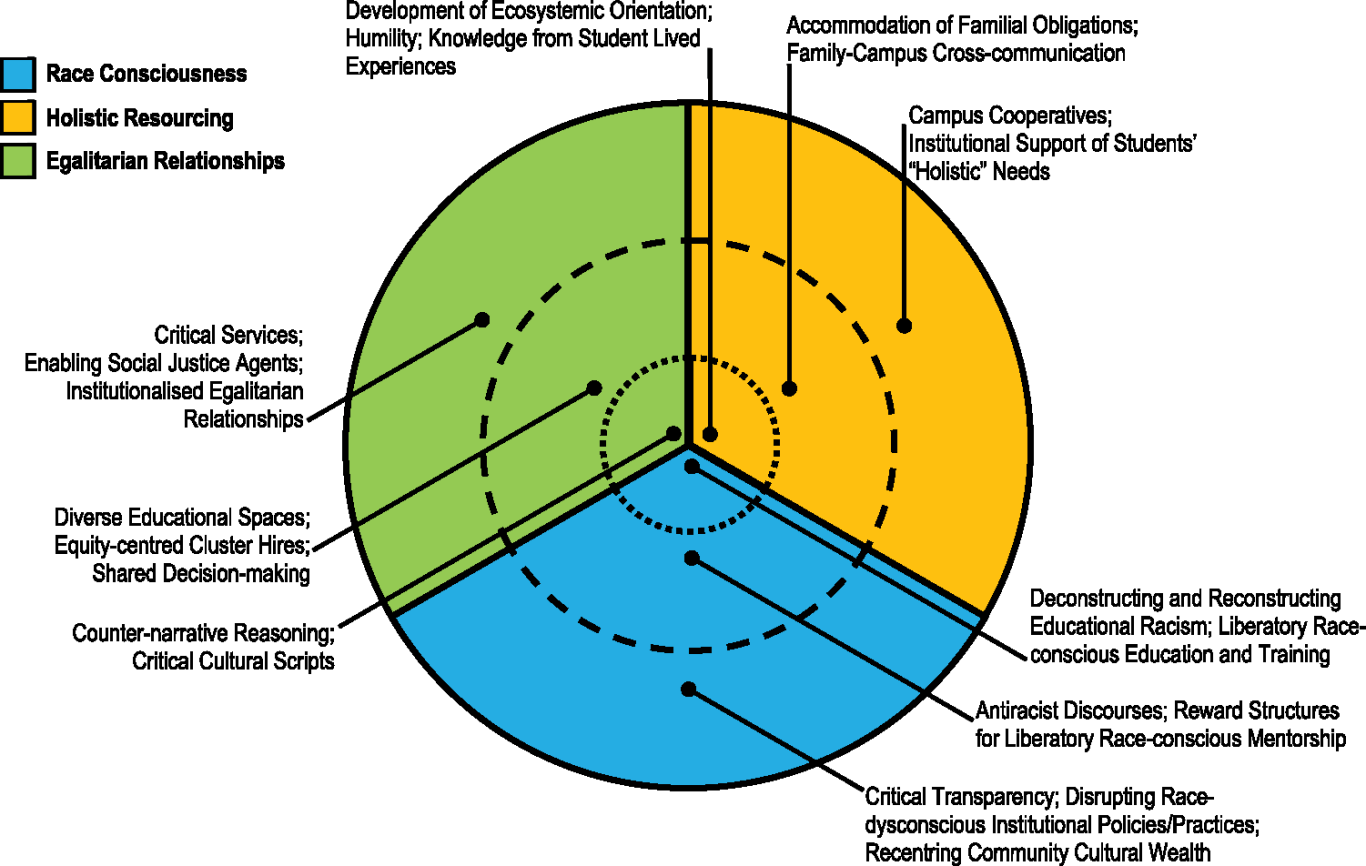
The three ecosystems of a non-hierarchical higher education institution. Inner Dotted Circle: Psychological Level. Middle Dashed Circle: Interpersonal Level. Outer Solid Circle: Institutional Level.

Dominant ideologies restrict the academic and life opportunities of intersectionally-marginalised students of colour (see Saetermoe et al., 2017; Stephens, Fryberg, et al., 2012; Vargas & Saetermoe, 2023; Vargas et al., 2021). Yet, it is possible to transform educational ecosystems and promote de-ideologisation (see also Bronfenbrenner, 1979; Lerner, 2015; Martín-Baró, 1994). Leaders must identify, challenge and eradicate dominant ideologies that maintain educational inequity. Leaders must also institutionalise culturally-congruent antiracist efforts. Thus, critical counter-ideologies are needed to mete out ecosystemic change (Figure 3).

### From race dysconsciousness to race consciousness

Vignette #1a (Race Dysconsciousness). Administrators are meeting to discuss a statewide policy that bans critical race theory from being taught on campus. An administrator of colour rejects the policy, explaining that critical race theory is helpful in understanding the endemic and interconnected nature of racism, white supremacy and capitalism. A white administrator retorts, ‘See, this is the kind of language that indoctrinates us to hate white people and our country. There’s no room for critical race theory on our campus and we need to put an end to it now’.

Leaders need time and courage to replace institutional policies and practices that align with a white supremacist past (Vargas & Saetermoe, 2023). Race-neutral admissions practices and colour-evading policies prioritise efficiency, as white-centric meritocratic ideas and deficit-based pedagogy go unchecked. Institutions reinforce practices that dismiss, or make invisible, the underlying explanations for educational inequity, thus enabling a hegemonic and ideologised reality (see also Martín-Baró, 1994). It is crucial to evaluate uncritical and racist ideas about what matters in education and, instead, support race consciousness within campuses. Vargas and Saetermoe argue that *race consciousness* affords a mechanism by which to resist ecosystemic racism and transform racist individual-systemic relations across all educational settings. This mechanism reflects an awareness and vigilance of the omnipresent and pervasive character of racial power. Race consciousness is a prerequisite of institutional and social norms that disrupt racist policies/practices and recentre students’ *community cultural wealth*, which describes the culture-specific skills, knowledges and perspectives employed by intersectionally-marginalised students of colour to survive within racist societies (see Yosso, 2005). Institutions can also lean on *critical transparency* and use mixed-methods and multi-sourced data collection activities to critique race-dysconscious policies and practices. Critical transparency demands input from experts in ethnic studies and fields that attract marginalised students (e.g., deaf, gender and queer studies). Critical transparency ought to unveil unflattering data about students, academics and their experiences. This assuages racial hostility and other forms of subordination. Rebuilding institutions involves confronting challenges, engaging in uncomfortable dialogues and applying practices as a collectivity.

Student success relies on campus cultures that nurture positive interpersonal academic-student relationships. Critical forms of mentorship mitigate the pushout problem (see Vargas et al., 2021). Unfortunately, inconsistencies in how campuses run mentorship programs make it difficult for students to reap the benefits of mentor-protégé relationships (see Law et al., 2020). This is especially true for intersectionally-marginalised students and academics of colour. There are no reward structures for academics of colour who mentor intersectionally-marginalised students of colour. Uncritical mentorship overburdens and harms academics of colour (e.g., slow tenure clocks), who mentor students of colour at higher rates than white academics. Mentorship takes time away from research and other related functions (e.g., publication). This invites the attrition of academics of colour and, in turn, fuels the social conditions that lead intersectionally-marginalised students to experience cultural mismatch in the first place (see Harper, 2012).

Student experiences are shaped by the psychology that educators bring into a relationship (Vargas et al., 2021). Campus personnel need to reframe their race-dysconscious assumptions and beliefs about racism through *liberatory race-conscious education* (see Vargas et al., in press). Education in liberatory race consciousness promotes social awareness and action via critical discussions about structural and institutional racism, the role of educators in building racial equity, and the need to recentre curriculum and pedagogy around the community cultural wealth of diverse students (Saetermoe et al., in press). Subordinated students who learn from (and with) educators living out antiracism can experience educational settings and norms that counter and eradicate ecosystemic racism. The scenario below provides one example:

Vignette #1b (Race Consciousness). Later in the meeting, another white administrator challenges the claim that critical race theory is anti-white. They offer historical context about the framework and discuss why leaders need to counter dominant racialised narratives that have impacted students of colour. This administrator projects a calm and assertive tone to ‘call in’ (versus ‘call out’) their colleagues. They ‘call in’ by helping others explore their understandings of critical race theory while providing constructive feedback. Although this approach does not change minds immediately, it does open doors for more meaningful reflection and dialogue.

### From academic capitalism to egalitarian relationships

Vignette #2a (Academic Capitalism). An email from the university chancellor expresses regret over a decision to raise tuition. ‘We know an increase in tuition will impact students, especially students of colour. Unfortunately, due to academic demands for higher wages, we need to uphold fiscal responsibility to maintain our competitive edge. Although there will be lay-offs and other cost-cutting measures to improve efficiency, we hold our obligation to ensure our students are able to have a high-quality educational experience’.

Higher education institutions steeped in academic capitalism operate in a hierarchical fashion. This harmful system prioritises economics and quantitative data-driven decision-making over the needs of people at the bottom of the hierarchy in order to achieve public-facing rankings based on white-centric markers of academic success (see also Harper, 2012). Marginalising pedagogies create unfair competitive cultures that represent a cultural mismatch. To draw upon campus strengths, there is a need for *egalitarian relationships*, or race-conscious interpersonal transactions that integrate the perspectives of marginalised students and academics (see also Yosso, 2005). Egalitarian relationships attenuate hierarchy and relational supremacy. With institutional support, these relationships open spaces wherein marginalised students and academics can become change agents who play active roles in the co-construction of culturally-congruent and equity-minded campuses. Concurrently, campuses are not designed to cultivate belonging among intersectionally-marginalised students of colour, which contributes to inequity in retention and graduation. To align campus culture and students’ experiences, Garcia (2019) argues in favour of modifying curricula, student services and institutional resources so that they enable students to become social justice agents. From a liberation lens (see Martín-Baró, 1994), the aim of higher education is to help students understand the legacies of oppression and the systems that must be overhauled, preferably in partnership with educators who hold comparable intersectionally-marginalised social statuses and identities. *Critical service* means educators critique academic capitalism as a central belief and act to replace policies, practices and campus cultures that lead to pushout. Institutions that truly serve are driven by student needs and not by rote monetary-based decision-making processes.

Academic capitalism ignores humanity and engenders untrusting interpersonal educator-student relationships that lead to student pushout. Subordinated students struggle to form genuine egalitarian relationships on campuses that lack critical masses of intersectionally-marginalised academics of colour (see Turner et al., 2017). Insufficient numbers of diverse academics obstruct routes to cultural match and solidarity. Equity-centred cluster hires are one effective strategy by which to diversify the pool of academics (Saetermoe et al., 2017). Diverse educational spaces can build connections for subordinated students and provide opportunities to organise and act. Moreover, egalitarian relationships create a sense of belonging and relationality in which like-minded people can heal, combine resources and build community.

Egalitarian relationships facilitate mindsets that are able to understand the ‘social space within which counter-hegemonic activity or contestation of dominant discourses can occur for both students and teachers [and administrators]’ (Garcia, 2019, p. 77). In remaking an institution with equal opportunities for discourse, actions against ecosystemic racism and cultural mismatch must incorporate counter-narratives (see also Vargas & Saetermoe, 2023; Vargas et al., 2021). Garcia argues that critical solutions draw from the strengths of subordinated students and academics. This allows all educators and students to overcome essentialised thoughts about merit, system-justification and individualism. Cultural scripts that send messages of belonging and that highlight the importance of social justice and giving back to one’s campus community can mitigate the pushout problem. The scenario below provides one example:

Vignette #2b (Egalitarian Relationships). In response to the chancellor, administrators and academics reach out to student leadership to discuss the significance of the email. Coming from a humanistic stance, all parties address their concerns through sincere and honest dialogue. They share their thoughts openly and build a relationship based on respect and trust. Administrators and academics practice power-sharing while critiquing how the email appears to pit students and academics against one another. All parties pose critical questions about the financial priorities of the university. They agree to develop a plan for a unified response to the chancellor.

### From rugged individualism to holistic resourcing

Vignette #3a (Rugged Individualism). Campus leaders in academic and student affairs are discussing the lasting effects of the pandemic on student learning, retention and graduation. One administrator highlights an increase in students’ depression and anxiety. Another administrator reviews a report about housing and food insecurity among students of colour. Afterwards, a third administrator states, ‘I know this may be unpopular but we all go through hard times. I know students have hardships but they also have to toughen up a bit and put in the work to graduate. We don’t want to create a culture of handholding and dependency. We also need to think about our finances. We can’t afford to pay for welfare-type services’.

Individualism reduces structural problems of society to personal problems of living (see Martín-Baró, 1994). Institutionalised rugged individualism puts intersectionally-marginalised students of colour in sink-or-swim contexts built for predominantly-white institutions and students. Rugged individualism encourages educators to focus solely on scholastic matters, thus ignoring the experiences of students who are unfamiliar with the ‘hidden curriculum’ needed to access basic and specialised resources that enhance their life opportunities. Intersectionally-marginalised students of colour are isolated, with little to no recourses to build a critical mass. Rugged individualism threatens academic persistence by forcing intersectionally-marginalised students of colour to assimilate to a system that does not recognise their community cultural wealth (see Yosso, 2005). Attending to holistic needs helps students persist. *Holistic resourcing* occurs when institutions systematically monitor and fulfil the social, economic, personal and cultural needs of subordinated students. Holistic resourcing means institutionalising processes that mutually benefit both students and campuses. For instance, campus cooperatives – devoted to computer programming, art designing, growing/serving food and other vital services – open pathways to graduation by giving students job experience through community-building activities that also meet practical and quotidian campus necessities.

Holistic resourcing requires accommodating the interpersonal needs of intersectionally-marginalised students of colour. Familial and other collectivistic values are key to subordinated students’ self-conceptualisation (Vargas & Kemmelmeier, 2013). Azaola (2020) found that familial support contributes to student persistence. Enhanced cross-communication between family and academia is especially beneficial to first-generation students, as role conflicts are diminished when the activities, triumphs and future prospects of successful higher education students are clarified and placed in both short- and long-term context (Groves & O’Shea, 2019).

It is difficult to manage and balance multiple institutional-, interpersonal- and individual-level interests. Educational leaders and academics who develop a critical orientation capable of addressing ecosystemic racism in education are in powerful positions to facilitate the success of intersectionally-marginalised students of colour (Vargas & Saetermoe, 2023). An ecosystemic orientation entails de-ideologising reality, humility, openness, rethinking essentialised practices and integrating marginalised people’s values (Martín-Baró, 1994; Yosso, 2005). Ecosystemic solutions alter how institutions welcome and educate students and how short- and long-term student outcomes are framed, in turn influencing students’ experiences and intrapsychic lives. Supported students experience acceptance, belonging, hope, resilience and success. Students’ experiences offer clues about how to critically assess programming strengths and weaknesses. The scenario below provides one example:

Vignette #3b (Holistic Resourcing). In response to the comment about ‘toughening up’, another highly-regarded administrator of colour interjects and shares their experience with homelessness when they were a student. ‘I can tell you that being homeless while being a student wasn’t easy. It was one of the toughest things I ever had to go through. I didn’t get to where I am today simply by being tough. I had educators who invested in me and connected me to essential resources that allowed me to emerge from homelessness. The people who helped me saw me as a whole person, flaws and all. They gave me the chance to thrive and end up in this meeting with you all today’.

## Discussion

Our modification of cultural mismatch theory demonstrates how the eradication of dominant ideologies and ecosystemic racism is contingent on institutional, interpersonal and intrapsychic processes that lean on antiracist tenets. This necessitates de-ideologising the racist ideological pillars of educational ecosystems (see also Bronfenbrenner, 1979; Martín-Baró, 1994). Absent multilevel solutions, intersectionally-marginalised students of colour will continue to experience cultural discontinuities that drive the pushout problem. Here, we return to Figure 3 to underscore broader ideas and exemplars that leaders may consider when developing, effecting and monitoring the efficacy of culturally-congruent equity-focused educational activities.

### Institutional considerations: eradicating dominant ideologies

Educational leadership can embrace bold and innovative visions by which to transform campus cultural norms and support intersectionally-marginalised students of colour. Such a transformation is contingent on the eradication of race dysconsciousness, academic capitalism and rugged individualism. Rather than operating as contented bystanders, leaders can advance race consciousness to disrupt traditional education and recentre community cultural wealth to engender cultural continuity for intersectionally-marginalised students of colour (see Yosso, 2005). Leaders can build campus cultures that value critical transparency and the positive impact of race conscious policies and practices. For instance, administrators can explicitly support liberatory race-conscious pedagogy trainings to deconstruct educational and institutional racism and reconstruct antiracist alternatives (see also Vargas et al., in press).

To create race-conscious cultures, the capitalist penchants of academia must be dismantled and replaced with non-hierarchical social arrangements. Egalitarian relationships require that campuses critically serve – rather than merely enrol – students represented by ethnic/racial institutional designations (e.g., Historically Black Colleges and Universities) that bring forth fiscal advantages (see also Garcia, 2019). Note that ethnic/racial designations provide access to specialised government grants or comparable monetary resources. Yet, these funds typically feed into superficial efforts towards promoting diversity, equity and inclusion (see also Pewewardy et al., 2018). Intersectionally-marginalised students of colour will be better served when leaders institutionalise reward structures to promote a culture of liberatory race-conscious pedagogy that enables students to become social justice agents inside and outside their campuses.

With proper apportionment of campus resources, intersectionally-marginalised students and academics of colour can benefit from social spaces where egalitarian relationships flourish, thus making institutions into a collectivity wherein the vestiges of rugged individualism are monitored, kept in check and channelled towards horizontal forms of individuality and respect for autonomy (see also Garcia, 2019; Vargas & Kemmelmeier, 2013). The call to action for leaders is to learn about and contend with novel antiracist aims that run against the capitalist-orthodoxies of vertically individualist higher educational systems. Holistic resourcing is one option by which to mitigate the effects of rugged individualism. Leaders can institutionalise holistic resourcing through campus cooperatives that allow students to work for housing, food, clothing, other basic needs and course credit. Financial strains are key pushout factors. Supporting basic needs offsets expenses and allows students to participate in the betterment of their campus (Avila et al., 2023). Overall, the de-ideologisation process for institutions depends on leaders who take risks.

### Interpersonal considerations: facilitating vital connections

Educational leadership must apply a critical lens to identify ecosystemic racism and its interpersonal roots (see Vargas & Saetermoe, 2023; Vargas et al., 2021). Left uninterrogated, the relational aspects of ecosystemic racism will derail the most well-intentioned antiracist projects. To counter race dysconsciousness, leaders can develop an ecosystemic orientation and broaden their notions of what ought to qualify as effective pedagogy. Such an orientation would see value in establishing reward structures to incentivise mentorship and other important student-academic relationships. Similar structures should exist to interrupt relational mechanisms that reproduce the status quo. The elimination of system-justifying discourses is one domain where leaders can invest institutional resources. Within student-academic interactions, system-justifying discourses will ‘shut down critical analysis about structural racism, ameliorate psychological distress, and restore legitimacy in the status quo’ (Vargas et al., 2021, p. 1050). System-justification leads to ineffective implementation of culturally-congruent relationship-building interventions. For this reason, Vargas and Saetermoe proposed antiracist counter-discursive strategies educators can leverage to combat system-justification. Antiracist counter-discourses are critical behavioural-communicative strategies based on race consciousness, historicisation, contextualisation, equity and other reconstructive canons, which can be useful when de-ideologising human relationships.

Leaders can apply an ecosystemic orientation when they create policies and norms that sustain diverse educational spaces for intersectionally-marginalised students of colour. One way to diversify educational spaces with student success in mind is *cluster hiring*, or practices that onboard multiple diverse academics to address campus diversity needs (Freeman, 2019). Leaders can use cluster hiring programs to hire diverse academics from various disciplines and identities, and who blend their pedagogical agendas to manifest culturally-congruent equity-focused educational environments (see Saetermoe et al., 2017; [UUH] Urban Universities for Health, 2015). Relatedly, diverse academics lead to more inclusive decision-making. In contrast to academic capitalism, leaders must work with subordinated students and academics to share decisions about curricula, capital investments, governance, mascot choices, building names and other cultural identifiers, as well as key rulings typically made in a clandestine top-down manner. Shared decision-making ensures that campus resources are strategically directed at combating racism rather than allowing these resources to be diluted by multiple well-intended but uncoordinated activities.

Cultural mismatch theory indicates how individualist learning environments hamper the academic prospects of marginalised students (see Stephens, Fryberg, et al., 2012; Stephens, Townsend, et al., 2012; Vasquez-Salgado et al., 2021). This is due to mismatches between the independent and competitive values of most campuses and the interdependent and collaborative values of students whose predecessors were once legally excluded from predominantly-white spaces. Attention to the cultural values of intersectionally-marginalised students of colour can offer clues about how to develop a culturally-congruent education experience from a relational stance. For instance, leaders can recognise and accommodate the needs of students with family or other obligations (Fuligni, 2007). Families aware of and invested in students’ higher education are able to remove barriers between family and campus life. Efforts to aid family-campus cross-communication should receive serious weight when transforming campus culture. Providing institutional support for students’ holistic needs via family-campus relationships may even inspire novel strategies and tools (e.g., community-campus partnerships) that leaders can utilise to build race-conscious campuses. The gains for students and educators (and communities) are limitless.

### Intrapsychic considerations: de-ideologising uncritical mindsets

The process of de-ideologising a culturally-incongruent institution can be psychologically taxing (see Vargas & Saetermoe, 2023). Leaders and academics should avoid *diversity fatigue*, or desensitisation towards doing diversity work (Smith et al., 2021). Diversity fatigue lessens the likelihood of participation in and support for diversity-related initiatives and is associated with the use of system-justifying discourses, especially among white educators (see also Vargas et al., 2021). Educators need support throughout all facets of race consciousness development (e.g., ongoing antiracism training). Vargas and Saetermoe liken race consciousness development to a lifelong journey in which knowledge about racism is complemented by deconstructing learned ideas of race, reconstructing the role of race in education, and enacting recentred values via collaborative and transparent efforts that mitigate inhumanities in education. The lives of intersectionally-marginalised students of colour and educators are brought into a syzygy with campus culture when educators internalise a new habitus grounded in critical and race-conscious pedagogical principles. When leaders integrate race-conscious attitudes and discourses in their own habitus, higher educational settings can become social spaces wherein both educators and students co-construct culturally-congruent education experiences.

Leaders must centre egalitarian relationships that reject academic capitalism and racist cultural scripts. Campuses and their leadership can lean on counter-ideologies as they devise ways to challenge the culturally-exploitive impact of academic capitalism. Leaders can model accountability through reparative efforts that address historical harms and cultural erasure (e.g., return of tribal remains and other sacred objects). In the same spirit, leaders can acknowledge the psychological harms of white supremacy and remove statues and building names that glorify colonisers, slaveholders and racists; actions towards reparations for descendants of enslaved persons should also be given serious weight (Perry & Barr, 2021).

In addition to symbolic actions, accountability requires humility and actionable steps to redress harm, build empowered culturally-congruent campuses and centre student experiences. Holistic resourcing involves applying the community cultural wealth brought to campuses by bilingual students, bicultural students, students with (grand)children, refugee students and otherwise marginalised students. Community cultural wealth can assist leaders and academics in developing new mental structures that help identify critical antiracist solutions to longstanding inequities. In the process, leaders embody accountability strategies that counter rugged individualism while modelling compassion, kindness and empathy.

### From ecosystemic considerations to ecosystemic actions

The road to educational justice is fraught with obstacles and pushback. Regardless of the social-political climate, educational leaders have a responsibility to foster a culturally-congruent education experience for intersectionally-marginalised students of colour. It is important to note that leaders are influenced by broader forces such as cultural racism, funding legislation, boards of trustees and donors. Despite these ecosystemic forces, latitude can be wielded when leaders implement antiracist actions within a campus. This article lists several ways to combat dominant ideologies across educational ecosystems. For educational leaders, it may be difficult to know where to commence the transformation process. Each campus has its own life and equity issues. The antiracist actions proposed in this subsection are proactive and intended to actualise a new vision of campus life. Higher education will not be transformed until leadership intentionally takes on educational inequity, not at the margins, but by restructuring education to best represent the fullness of the human condition (Tevis & Foste, 2023). Transforming higher education in this manner humanises all parties involved while also giving nations an edge with respect to diverse ideas, innovations and global contributions. Leaders can begin with a low-level adoption of our recommended considerations or, if human and economic resources allow, begin at a much larger scale. In either case, three guidelines are germane: collaborative problem-solving, ecosystemic change efforts and reflexivity.

To build a culturally-congruent education culture, the social methods that eschew white supremacist histories and policies cannot lay solely in the hands of leaders who have normalised the status quo (Chun & Feagin, 2022). De-ideologising ecosystemically racist institutions is a collaborative problem-solving endeavour. A valuable campus resource is the intelligentsia of intersectionally-marginalised academics and students of colour. Scholars in ethnic studies and antiracism are key to collaborative problem-solving, as they are best equipped to help leaders attend to unjust power dynamics (Walters et al., 2019). Leaders can humbly examine their own campus equity issues using tools like the Equity Scorecard (see Bensimon et al., 2012) and follow such needs assessments with transparent communication about persistent inequities. Assessments should also involve the collation and dissemination of institutional equity data. Collaborative problem-solving permits multiple and diverse campus stakeholders to formulate strategic solutions. Proposed changes in policies, procedures and practices can be contextualised by leaders who possess a ‘big-picture’ view of higher educational systems. Together, campus stakeholders should propose solutions that account for the dominant ideologies that must be toppled to create an antiracist culture.

The elimination of cultural discontinuities between white-centric academic spaces and the community cultural wealth of intersectionally-marginalised students of colour rests on efforts to dismantle ecosystemic racism in education (see also Vargas & Saetermoe, 2023; Vargas et al., 2021). Ecosystemic racism operates at the individual, interpersonal and institutional spheres of social life. At the individual level, educational leaders can serve as role models by publicising and supporting ongoing antiracism efforts. Leaders have to routinely learn about racism, neither ignore nor tolerate discrimination and prevent new discriminatory policies and practices from being executed (Chun & Evans, 2018). The eradication of enduring dominant ideologies also involves an interpersonal dimension. Campus cultures are defined by and shape the complex relationships among leaders, academics, staff and students. Ideally, all members of a campus should be encouraged to participate in antiracism education and discourses that emphasise not only interpersonal racism (e.g., implicit bias; stereotypes) but also the structural apparatuses (e.g., laws; norms) that assemble the backdrop for racist interpersonal transactions (see Al Shaibah, 2023). Leaders can assist a campus’s entrée to antiracism through clear and inviting statements about how campus culture can improve through antiracism. Institutional-level action is also paramount. Antiracist campuses acknowledge how mentorship is pivotal to the success of intersectionally-marginalised students of colour. Leaders can support policies that incentivise liberatory race-conscious mentorship, which should be considered in decisions about academics’ retention, tenure and promotion. Additionally, incentivising the rewriting of curricula, as well as grassroots-level pedagogy planning, warrant similar leadership support.

Reflexivity is crucial. Campus stakeholders must meet regularly to discuss the impact of policy implementations on antiracist efforts. It is common for antiracist educators to encounter hostile backlash or collective apathy (see Pewewardy et al., 2018; Vargas & Saetermoe, 2023). In these instances, educational leaders must stand behind those being challenged (Martin et al., 2022). Getting stakeholders to deliberate what a campus transformation should entail requires spaces where people can explore their own experiences with race/racism. If done incorrectly or superficially, deconstructing prior beliefs rooted in dominant ideologies can interfere with a wholehearted acceptance of antiracist praxis. Another effective reflexive approach is to host speakers and organisers who can impart critiques of an institution’s theory of change. Antiracist experts exist both in and out of the campus setting. With the backing of institutional leadership, the valuable knowledge of antiracist experts can facilitate the formative and difficult periods of de-ideologisation and liberational transformation.

### Final thoughts: differential risks, common social-political realities

Educational leaders are not all created equal. Becoming race conscious and antiracist introduces distinct personal and interpersonal risks for intersectionally-marginalised leaders of colour and their white counterparts (see Vargas & Saetermoe, 2023). Moreover, institutional-level antiracist work often produces backlash. Yet, even among white leaders who have rejected whiteness and white supremacy, vulnerability to racial hostility and retribution is less vis-à-vis marginalised leaders’ own risks. To be clear, the burden of excising white supremacy from educational ecosystems falls onto white leaders (see Vargas et al., 2021). As such, white leaders must collaborate with less risk-prone leaders of colour to protect and support the antiracist agendas of more risk-prone leaders, academics and students of colour.

Regardless of identity or social status, educational leaders participate in their nation’s social-political ecosystem. Social politics can explain legislative efforts against antiracist-based education. In the US, laws designed to ban critical race theory and similar perspectives have less to do with legitimate injuries related to diversity, equity and inclusion and more to do with right-wing efforts to privatise public education sectors and redirect tax dollars towards for-profit private and religious schools (see Morgan, 2022). Laws prohibiting antiracist-based instruction in K-12 public education will yield inequitable outcomes for intersectionally-marginalised students of colour (see Kaerwer & Pritchett, 2023). Higher education systems are under similar attack (see Miller et al., 2023). To safeguard public education from privatisation, leaders will have to work together within legal arenas to counter bans against antiracist-based pedagogies. Although these bans are flagrant violations of the First and Fourteenth Amendments of the US Constitution, litigators will need evidence to articulate the harms caused by specific statutes (see Gutzmann, 2021). Leaders must unite within campuses, across campus districts, and throughout entire higher education systems. In line with Gutzmann, leaders and litigators can commission empirical studies that detail the harms incurred by intersectionally-marginalised students of colour as a consequence of racist educational laws and policies. Data about chilling effects experienced by academics of colour (e.g., barring of books or historical topics) will also be instrumental. Evidence-based legal arguments can then be constructed to demonstrate both the unconstitutionality of anti-antiracism legislation and the real threats these laws pose to students’ critical reasoning skills and opportunities to thrive in a diverse and globalised world.

## Conclusion

Educational leadership has a responsibility to identify and uproot the white-centric ideologies that foster ecosystemic racism in educational spaces. In the US and other Western nations, dominant ideologies – shaped by the interconnected nature of contemporary social, political and economic realities – produce cultural discontinuities that harm intersectionally-marginalised students and academics of colour. Understanding how race dysconsciousness, academic capitalism and rugged individualism (or other nation-specific ideologies) operate within the ecosystems of higher education is a critical step towards policies and practices that disrupt the current state of affairs. More important than mere understanding is action. Leaders have to confront challenges, engage in difficult dialogues and apply critical collective practices that promote belonging and cultural congruence for intersectionally-marginalised students and academics of colour. De-ideologised collective practices integrate the values of subordinated students and academics so as to co-construct egalitarian relationships, institutionalise shared decision-making and incorporate community cultural wealth. Critical educational leadership is driven by student needs and not by economic-based decision-making processes. This style of antiracist leadership is key to transforming predominantly-white institutions into culturally-congruent educational spaces. Liberational transformations centralise holistic education and student-focused reparative efforts that address historical injuries, cultural erasure and academic pushout. The time to lead this radical transformation is now!
